# The Association between Insulin Resistance and Cardiovascular Disease Risk: A Community-Based Cross-Sectional Study among Taiwanese People Aged over 50 Years

**DOI:** 10.3390/ijerph17197195

**Published:** 2020-10-01

**Authors:** Mei-Chun Lu, Wei-Ching Fang, Wen-Cheng Li, Wei-Chung Yeh, Ying-Hua Shieh, Jau-Yuan Chen

**Affiliations:** 1 Department of Family Medicine, Chang-Gung Memorial Hospital, Linkou Branch, Taoyuan City 33305, Taiwan; b101092026@tmu.edu.tw (M.-C.L.); winds75526@gmail.com (W.-C.F.); wcl20130714@gmail.com (W.-C.L.); sendoh777777@gmail.com (W.-C.Y.); 2 Department of Family Medicine, Chang Gung Memorial Hospital, Taoyuan Branch, Taoyuan City 33378, Taiwan; 3 College of Medicine, Chang Gung University, Taoyuan City 33302, Taiwan; 4 Department of Health Management, Xiamen Chang-Gung Hospital, Xiamen 361000, China; 5 Department of Family Medicine, New Taipei Municipal TuCheng Hospital, New Taipei City 23652, Taiwan; tony470120@gmail.com

**Keywords:** insulin resistance, HOMA-IR, prediction, CVD, Framingham risk score

## Abstract

Background and Aims: Previous studies have implied that insulin resistance (IR) could represent a major underlying abnormality leading to cardiovascular disease (CVD). The aim of this study was to evaluate the relationships between IR (estimated by the homeostasis model assessment of IR (HOMA-IR) index) and CVD risk among middle-aged and elderly Taiwanese individuals. Methods: In this cross-sectional, community-based study, a total of 320 participants were interviewed to collect demographical parameters and blood samples. The recruited participants were divided into tertiles according to their levels of HOMA-IR. The Framingham risk score (FRS) was calculated according to the 2008 general CVD risk model from the Framingham Heart Study. Results: The HOMA-IR index was significantly correlated with the FRS, with a Pearson’s coefficient of 0.22. In the multiple logistic regression model, a higher HOMA-IR level was significantly associated with a high FRS (FRS ≥ 20%) (highest tertile vs. lowest tertile of HOMA-IR, crude OR = 3.69; 95% CI = 1.79–7.62), even after adjusting for smoking, fasting plasma glucose (FPG), and systolic blood pressure (SBP) (highest tertile vs. lowest tertile of HOMA-IR, adjusted OR = 11.51; 95% CI = 2.55–51.94). The area under the receiver operating characteristic curve for the HOMA-IR index as the predictor of high FRS was 0.627, and the optimal HOMA-IR cutoff value was 1.215 (sensitivity = 83.6%, specificity = 42.9%). Conclusions: We considered that HOMA-IR is an independent factor but that it cannot be used solely for evaluating the CVD risk due to the low AUC value. Further prospective cohort studies are warranted to better assess the relationship between CVD risk and insulin resistance.

## 1. Introduction

Insulin resistance (IR) is thought to be the key mechanism of metabolic syndrome, a cluster of cardiovascular risk factors [1,2,3,4,5,6]. Previous studies have shown that hyperinsulinemia was an independent predictor of incident myocardial ischemic events in a non-diabetic population [7]. IR is also an independent risk factor for various chronic diseases, such as hypertension [8], hepatic steatosis and steatohepatitis [9], chronic kidney disease [10], and even lung cancer [11] and Alzheimer’s disease [12].

Overnutrition, obesity, and specific dietary components can trigger IR [9,13]. Excess daily calories, fructose, and saturated fatty acid intake; a lack of n-3 polyunsaturated fatty acids and antioxidants; as well as intestinal microbiome imbalance can increase the oxidative stress in the liver and lead to IR and non-alcoholic hepatic steatosis and steatohepatitis [9].

The hyperinsulinemic–euglycemic clamp technique is the gold standard for evaluating insulin resistance, but it is impractical in clinical practice. Thus, surrogate markers such as the homeostasis model assessment of IR (HOMA-IR) have been developed to assess IR [14]. In Taiwan, the 2008 version of Framingham risk score (FRS) is frequently used by clinicians to estimate a person’s risk of cardiovascular disease (CVD) in the next 10 years. Additionally, previous studies have shown that the FRS is applicable in Asian populations [15]. However, only limited studies have discussed the association of HOMA-IR with FRS [16,17]. For example, obese children with an increased HOMA-IR have a higher CVD risk [17].

Therefore, the aim of the current study was to investigate the associations between the HOMA-IR index and FRS and evaluate the ability of HOMA-IR to identify community residents with a high FRS (FRS ≥ 20%) in middle-aged and elderly Taiwanese individuals.

## 2. Materials and Methods

### 2.1. Participants

This was a cross-sectional study. The participants were recruited through a community health promotion project in Linkou Chang Gung Memorial Hospital in Taiwan from February 2014 to August 2014. The project was approved by the Institutional Review Board (102-2304B) of Linkou Chang Gung Memorial Hospital, and informed consent was signed by all the participants before enrollment.

A total of 400 participants aged over 50 years living in Guishan district (a rural district in northeastern Taiwan) were recruited. General information, including medical history and lifestyle, was obtained by appropriately trained interviewers. Participants with a history of CVD (*n* = 19), aged 75 years and above (*n* = 56), or with incomplete data (*n* = 3) were excluded. Extreme outliers of HOMA-IR (*n* = 2) were excluded. Finally, 320 participants were included in this study.

### 2.2. Anthropometric

Detailed anthropometric measurements, such as height, weight, waist circumference (WC), and blood pressure, were conducted by trained research assistants or nurses under the supervision of a medical doctor. The measurement of heights was carried out with the subjects standing with their feet together. Body mass index (BMI) was calculated by dividing weight by the square of the height (kg/m^2^). Blood pressure (BP) was measured using an automated sphygmomanometer after the participants were relaxed and seated for more than 10 min. The WC was measured halfway between the iliac crest and the lowest rib at the end of a relaxed expiration. The height meter and weight scale were calibrated once per day.

### 2.3. Laboratory Examinations

Participants were requested to fast for at least 12 h and avoid a high-fat diet or alcohol consumption before blood sampling. Blood samples were stored in a refrigerator at 4 °C and then sent to the hospital laboratory. Clinical biochemistry tests were performed in a hospital laboratory accredited by the College of American Pathologists. The fasting plasma glucose (FPG); fasting insulin; alanine aminotransferase (ALT); creatinine; uric acid; and fasting lipid profiles, such as triglyceride (TG), high-density lipoprotein cholesterol (HDL-C), and low-density lipoprotein cholesterol (LDL-C), were measured.

### 2.4. Assessment of Insulin Resistance

We assessed insulin resistance by HOMA-IR [14], which is expressed as:

$$\mathrm{HOMA}-\mathrm{IR}=\;\frac{\mathrm{fasting}\;\mathrm{glucose}\;\left(\mathrm{in}\;\mathrm{mmol}/L\right)\times \;\mathrm{fasting}\;\mathrm{insulin}\;\left(\mathrm{in}\;\mathrm{mU}/\mathrm{ml}\right)}{22.5}$$

Subjects were divided into tertiles based on the HOMA-IR index:

Low HOMA-IR group: HOMA-IR ≤ 1.15;

Middle HOMA-IR group: 1.15 < HOMA-IR ≤ 1.93;

High HOMA-IR group: HOMA-IR > 1.93.

### 2.5. Cardiovascular Risk Assessments

We used the 2008 Framingham risk score (FRS) assessments to determine the risk of CVD [18].

The risk factors and the equations used to calculate the FRS are listed below:

Women

$$\begin{array}{c} \begin{array}{ll} \mathrm{Risk}\;\mathrm{Factors}= & \;\left(\ln\left(\mathrm{Age}\right)\times 2.32888\right)+\left(\ln\left(\mathrm{Total}\;\mathrm{cholesterol}\right)\times 1.20904\right)\\ & -\left(\ln\left(\mathrm{HDL}\;\mathrm{Cholesterol}\right)\;\times 0.70833\right)\\ & +\left(\ln\left(\mathrm{Systolic}\;\mathrm{blood}\;\mathrm{pressure}\right)\;\times \;\mathrm{blood}\;\mathrm{pressure}\;\mathrm{medication}\;\mathrm{factor}\right)\\ & +\mathrm{Cigarette}\;\mathrm{smoker}\;\mathrm{factor}+\mathrm{Diabetes}\;\mathrm{present}\;\mathrm{factor}-26.1931 \end{array}\\ \begin{array}{c} \mathrm{FRS}=100\times \;\left({\left(1-0.95012\right)}^{r}\right)\\ r={\;e}^{\left(\mathrm{Risk}\;\mathrm{factors}\right)} \end{array} \end{array}$$

Men

$$\begin{array}{c} \begin{array}{ll} \mathrm{Risk}\;\mathrm{Factors}= & \;\left(\ln\left(\mathrm{Age}\right)\times 3.06117\right)+\left(\ln\left(\mathrm{Total}\;\mathrm{cholesterol}\right)\times 1.12370\right)\\ & -\left(\ln\left(\mathrm{HDL}\;\mathrm{Cholesterol}\right)\;\times 0.93263\right)\\ & +\left(\ln\left(\mathrm{Systolic}\;\mathrm{blood}\;\mathrm{pressure}\right)\;\times \;\mathrm{blood}\;\mathrm{pressure}\;\mathrm{medication}\;\mathrm{factor}\right)\\ & +\mathrm{Cigarette}\;\mathrm{smoker}\;\mathrm{factor}+\mathrm{Diabetes}\;\mathrm{present}\;\mathrm{factor}-23.9802 \end{array}\\ \begin{array}{c} \mathrm{FRS}=100\times \;\left({\left(1-0.88936\right)}^{r}\right)\\ r={\;e}^{\left(\mathrm{Risk}\;\mathrm{factors}\right)} \end{array} \end{array}$$

A high FRS was defined as an FRS ≥ 20%.

### 2.6. Statistical Analysis

Demographics and clinical characteristics are presented as means with standard deviations and counts with percentages for continuous and discrete variables, respectively. Descriptive statistics were presented, and differences between groups with different HOMA-IR levels were compared using one-way ANOVA for continuous data and the chi-square test for categorical data. The correlation of cardiovascular disease risk factors and HOMA-IR levels was assessed using Pearson’s analysis. Multiple logistic regression analysis was used to adjust for covariates. A receiver operating characteristic (ROC) curve was constructed to assess the performance of the HOMA-IR to predict a high FRS. Youden’s index, which maximizes the sum of sensitivity and specificity (max (sensitivity+ specificity-1)), was used to determine the optimal cutoff point, and the corresponding sensitivity, specificity, and area under the receiver operating characteristic curve (AUC) were calculated. All the statistical tests were regarded as statistically significant, with a *p*-value less than 0.05 (two-sided). Data were analyzed using the Statistical Package for the Social Sciences software, version 22.0 (SPSS Inc., Chicago, IL, USA).

## 3. Results

### 3.1. Baseline Characteristics of the Patient Population

**Figure 1:**
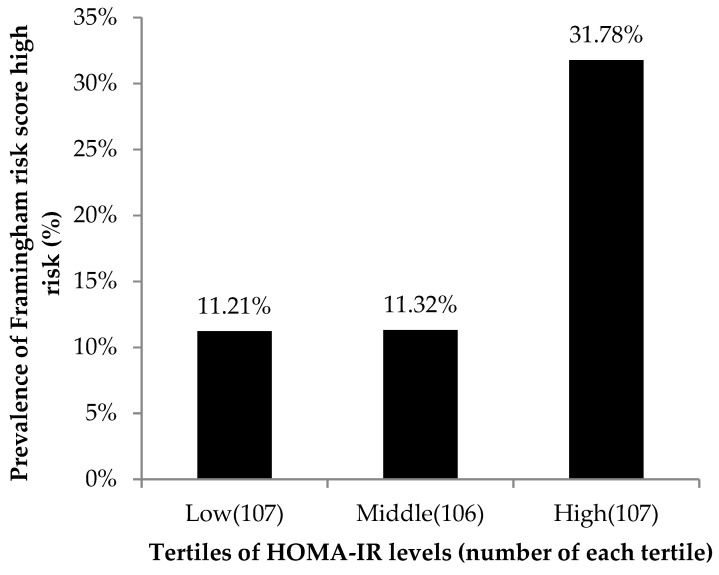
Prevalence of high FRS (FRS ≥ 20%) according to the tertiles of the HOMA-IR index levels. The prevalence of high FRS was higher among the high HOMA-IR tertile than among the two lower tertiles.

The general characteristics of the participants according to the tertile of the HOMA-IR level are summarized in Table 1. Overall, the mean age was 61.88 ± 6.21 years, and the mean FRS was 14.31 ± 11.47%. There were no significant differences between the three groups concerning age, creatinine, smoking, alcohol drinking, and regular exercise. However, the mean FRS was significantly higher in the groups with higher HOMA-IR levels. The mean FRS were 10.95 ± 7.85% (low HOMA-IR group), 13.94 ± 10.21% (middle HOMA-IR group), and 18.04 ± 14.33% (high HOMA-IR group), respectively. Other parameters with significant differences between the three groups with *p*-values lower than 0.05 were SBP, BMI, WC, ALT, FPG, HDL-C, TG, LDL-C, and uric acid. Figure 1 shows that the prevalence of high FRS was higher among the high HOMA-IR tertile than among the two lower tertiles (*p*-value for the Cochran–Armitage trend test of <0.001).

### 3.2. Correlation Analysis

**Figure 2:**
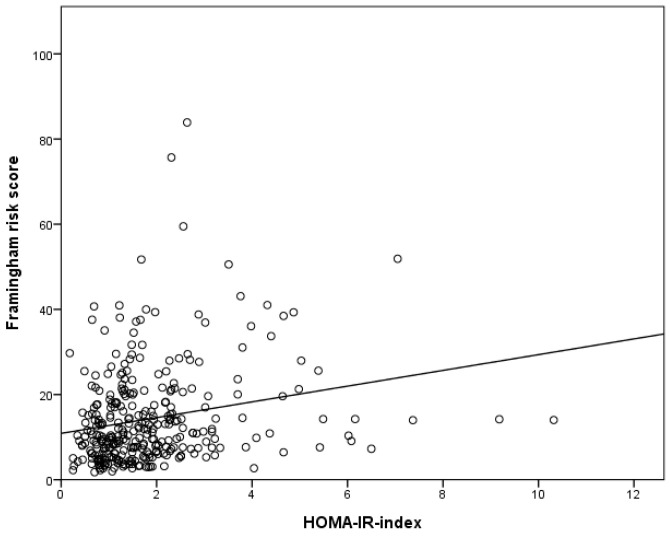
Correlation coefficients between the HOMA-IR levels and the Framingham risk score.

The correlations of the HOMA-IR levels with the CVD risk factors are shown in Table 2. The factors with significant positive correlations with IR were SBP, BMI, FRS, WC, FPG, TG, and uric acid, while HDL-C and LDL-C showed significant negative correlations with IR. Among them, BMI, WC, FPG, and TG showed a stronger correlation with IR than the correlation of the FRS with IR. Considering that the negative correlation of LDL-C was very weak (Pearson’s coefficient -0.11), we did not involve LDL-C in the subsequent multiple logistic regression analysis. Figure 2 demonstrates the correlation of FRS and IR with a Pearson’s coefficient of 0.22.

### 3.3. Association between the Tertiles of HOMA-IR Levels and the Framingham Risk Factors

Table 3 shows the results of the multiple logistic regression analyses in comparing the risk of high FRS between subjects with different HOMA-IR levels (with the low HOMA-IR level group as the reference group). In Model 1 (unadjusted), the odds ratios (ORs) for the middle and high HOMA-IR groups were 2.71 (95% CI: 1.29–5.69, *p*-value 0.009) and 3.69 (95% CI: 1.79–7.62, *p*-value < 0.001), respectively. In Model 2, the ORs (95% CI) were determined after further adjustment for gender. In Model 3, the ORs (95% CI) were determined after further adjustment for gender, age, and BMI. In Model 4, the HOMA-IR level remained significantly correlated with high FRS after adjustment for covariates, including age, gender, BMI, smoking status, SBP, and FPG. The adjusted ORs for the middle HOMA-IR and high HOMA-IR groups were 11.31 (95% CI: 2.94–43.52, *p*-value < 0.001) and 11.51 (95% CI: 2.55–51.94, *p*-value = 0.001), respectively.

### 3.4. ROC Analysis

**Figure 3:**
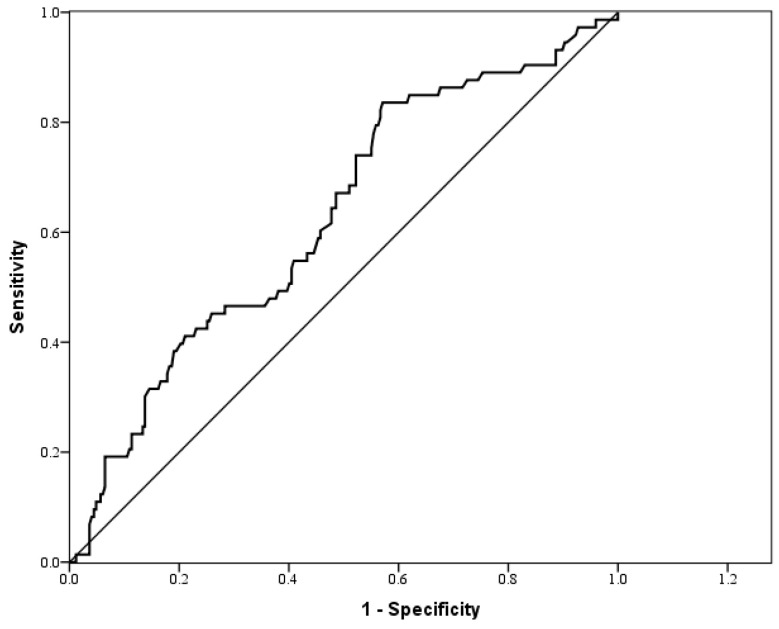
ROC curve for the HOMA-IR index as a predictor of the Framingham risk score.

The AUC for the HOMA-IR index as a predictor of high FRS was 0.627, as shown in Table 4 and Figure 3. According to Youden’s index, the optimal cutoff point for the HOMA-IR index for predicting a high FRS was 1.215, with a sensitivity and a specificity of 83.6% and 42.9%, respectively.

## 4. Discussion

Our study objective was to investigate the associations between IR and future CVD risk. In this community-based cross-sectional study among Taiwanese people aged over 50 years, the level was positively associated with the FRS after adjusting for potential confounders. Several studies have reported a positive correlation between CVD risk and IR [19,20,21,22], which is consistent with our findings.

HOMA-IR is a frequently used tool to estimate IR. However, there are only limited studies focusing on the use of the HOMA-IR level for predicting high FRS. In our study, participants with high HOMA-IR levels had a higher prevalence of high FRS compared with those with lower HOMA-IR levels (Figure 1). After adjusting for other risk factors, including sex, age, BMI, current smoking status, FPG, and SBP through multiple logistic regression models, the HOMA-IR level was still significantly associated with high FRS. These results reinforce that HOMA-IR is an independent risk factor for high FRS. We then evaluated the ability of HOMA-IR to identify community residents with a high FRS. However, due to the low specificity and low AUC value, HOMA-IR cannot be used as the sole predictor of high FRS.

The recent studies have revealed that there are several mechanisms to induce IR, such as genetic defects, overnutrition, obesity, and inflammation [13,23]. In overnutrition status, the excess intra-cellular free fatty acids will lead to the activation of PKC and NFκB, then induce serine 307 phosphorylation for IRS-1, which inhibits the insulin signal pathway and eventually results in IR in the skeleton muscle and hepatocytes. The PKC activation and increased free fatty acid flux to the liver results in hepatosteatosis [13,24,25].

Excess free fatty acids in cardiomyocytes and endothelial cells also cause serine 307 phosphorylation for IRS-1, then inhibit the insulin signaling pathway [23,26]. An inhibited insulin signaling pathway results in increased free fatty acid oxidation and decreased glucose oxidation in cardiomyocyte and causes cell death [23]. In the endothelial cells, IR causes an imbalance between the production of eNO and the secretion of endothelin-1, resulting in endothelial dysfunction [26].

The early detection of IR, as well as interventions in lifestyle and dietary components, could decrease the development and progression of IR and reverse further damage to the liver, heart, and endothelium. In Taiwan, free adult health exams offered by the Health Promotion Administration cover all the needed parameters for FRS calculation and fasting glucose, but not fasting insulin. Besides, clinicians do not routinely check HOMA-IR. For individuals with diabetes or obesity, it is easy to link their condition with insulin resistance. However, for high FRS individuals who are elderly, smokers, or have hypertension, it is harder for clinicians to think about IR. This study revealed that HOMA-IR is an independent risk factor for high FRS. Clinicians should be more alert for IR in high-FRS individuals, and offer lifestyle and dietary interventions earlier for better prognosis.

Lifestyle interventions and dietary interventions are reported to improve IR, the lipid profile, and liver function enzymes [9,13]. Lifestyle interventions include body weight loss of over 5% of the initial weight, physical activity for 150 min/week or more, training exercises for four weeks, or a regular exercise program. Dietary interventions include daily calorie restriction by reducing 500 Kcal from usual food consumption, a high-protein diet, fructose restriction, and choosing a Mediterranean diet. Supplementation with anti-oxidatives, amino acids, vitamins, n-3 polyunsaturated fatty acids, and prebiotics/probiotics may have potential benefits, but lack sufficient evidence [9].

This study still has some limitations. First, the study was cross-sectional; therefore, it is unable to explore the cause–effect relationship between FRS and IR. Second, we do not have a direct outcome measure of CVD. Third, we did not check Hba1c, a more reliable marker for identifying pre-diabetic and diabetic individuals. Finally, our sample size was relatively small. Further studies are warranted with unknown confounders.

## 5. Conclusions

In conclusion, a high FRS (FRS ≥ 20%) was significantly associated with IR in the middle-aged and elderly population in Taiwan. HOMA-IR is an independent risk factor for high FRS, but cannot be used as the sole predictor due to its low specificity. Further prospective cohort studies are warranted to better assess the relationship between CVD risk and insulin resistance in middle-aged and elderly Taiwanese populations.

## Figures and Tables

**Table 1:** General characteristics of the study population according to the tertiles of the HOMA-IR levels.

	HOMA-IR Levels								
	Total		Low (HOMA-IR ≤ 1.15)		Middle (1.15 < HOMA-IR ≤ 1.93)		High (HOMA-IR > 1.93)		
Variables	(*n* = 320)		(*n* = 107)		(*n* = 106)		(*n* = 107)		*p*-value
Age (year)	61.88	±6.21	61.38	±6.13	61.72	±6.54	62.53	±5.93	0.38
SBP (mmHg)	128.76	±16.09	124.52	±16.77	129.20	±14.41	132.56	±16.11	0.001
DBP (mmHg)	78.12	±10.80	76.80	±11.56	78.01	±9.97	79.55	±10.72	0.176
BMI (kg/m^2^)	24.61	±3.52	22.95	±2.99	24.55	±2.82	26.32	±3.84	<0.001
Waist circumference (cm)	84.54	±9.37	80.31	±7.52	83.62	±8.04	89.68	±9.89	<0.001
ALT (U/L)	22.89	±12.89	19.61	±7.49	22.18	±11.71	26.89	±16.75	<0.001
Creatinine (mg/dL)	0.72	±0.28	0.69	±0.16	0.73	±0.32	0.74	±0.34	0.37
eGFR (ml/min/1.73m^2^)	117.43	±30.65	121.03	±28.94	116.23	±31.53	115.01	±31.37	0.32
FPG (mg/dL)	95.33	±21.96	85.065	±8.44	92.113	±12.37	108.77	±30.49	<0.001
HDL-C (mg/dL)	54.96	±13.83	61.037	±14.67	54.472	±12.36	49.36	±11.84	<0.001
HOMA-IR index	1.85	±1.36	0.80	±0.23	1.51	±0.23	3.24	±1.52	<0.001
LDL-C (mg/dL)	120.20	±31.85	121.77	±34.23	124.78	±30.51	114.10	±29.96	0.04
Triglyceride (mg/dL)	123.37	±65.14	93.97	±41.90	119.44	±54.73	156.66	±77.71	<0.001
Uric Acid (mg/dL)	5.71	±1.41	5.39	±1.30	5.71	±1.41	6.03	±1.45	0.003
Framingham risk score (%)	14.31	±11.47	10.95	±7.85	13.94	±10.21	18.04	±14.33	<0.001
Current smoking, *n* (%)	35	(10.9)	13	(12.1)	10	(9.4)	12	(11.2)	0.81
Alcohol drinking ≥ 2 times/week, *n* (%)	62	(19.4)	26	(24.3)	21	(19.8)	15	(14.0)	0.16
Regular exercise, *n* (%)	259	(80.9)	92	(86.0)	87	(82.1)	80	(74.8)	0.11
HTN, *n* (%)	152	(47.5)	40	(37.4)	42	(39.6)	70	(65.4)	<0.001
DM, *n* (%)	54	(16.9)	2	(1.9)	13	(12.3)	39	(36.4)	<0.001
Hyperlipidemia, *n* (%)	211	(65.9)	60	(56.1)	70	(66.0)	81	(75.7)	0.01

Notes: Clinical characteristics are expressed as the mean ± SD for continuous variables and *n* (%) for categorical variables. *p*-values were derived from a one-way analysis of variance (one-way ANOVA) for continuous variables and chi-square tests for categorical variables. Abbreviations: BMI, body mass index; SBP, systolic blood pressure; DBP, diastolic blood pressure; ALT, alanine aminotransferase; FPG, fasting plasma glucose; HDL-C, high-density lipoprotein cholesterol; LDL-C, low-density lipoprotein cholesterol; TG, triglyceride; HTN, hypertension.

**Table 2:** Pearson correlation coefficient of the HOMA-IR levels with the cardiovascular disease risk factors.

	HOMA-IR Index (*n* = 320)	
Variables	Pearson’s coefficient	*p*-value
Age (year)	0.02	0.68
SBP (mmHg)	0.16	0.005
DBP (mmHg)	0.09	0.126
BMI (kg/m^2^)	0.46	<0.001
Framingham risk score (%)	0.22	<0.001
Waist circumference (cm)	0.45	<0.001
FPG (mg/dL)	0.58	<0.001
HDL-C (mg/dL)	−0.32	<0.001
TG (mg/dL)	0.34	<0.001
LDL-C (mg/dL)	−0.11	0.04
Uric Acid (mg/dL)	0.16	0.003

Abbreviations: SBP, systolic blood pressure; DBP, diastolic blood pressure; BMI, body mass index; FPG, fasting plasma glucose; HDL-C, high-density lipoprotein cholesterol; LDL-C, low-density lipoprotein cholesterol; TG, triglyceride.

**Table 3:** Association between the tertiles of the HOMA-IR levels and a high FRS (FRS ≥ 20%).

	Model 1			Model 2			Model 3			Model 4		
Variables	OR	(95% CI)	*p*-value	OR	(95% CI)	*p*-value	OR	(95% CI)	*p*-value	OR	(95% CI)	*p*-value
Low	1.00			1.00			1.00			1.00		
Middle	2.71	(1.29–5.69)	0.009	5.16	(2.12–12.57)	<0.001	5.66	(2.17–14.78)	<0.001	11.31	(2.94–43.52)	<0.001
High	3.69	(1.79–7.62)	<0.001	9.67	(3.83–24.37)	<0.001	9.01	(3.14–25.81)	<0.001	11.51	(2.55–51.94)	0.001
*p*-value for trend			<0.001			<0.001			<0.001			0.002

Model 1: unadjusted; Model 2: adjusted for sex; Model 3: adjusted for factors in model 2 plus age and BMI; Model 4: adjusted for factors in model 3 plus smoking, FPG, SBP. Abbreviations: BMI, body mass index; FPG, fasting plasma glucose; SBP, systolic blood pressure; CI, confidence interval.

**Table 4:** The areas under the ROC curve (AUC), sensitivity, and specificity of the optimized cut-off points for the HOMA-IR index in predicting high FRS (FRS ≥ 20%).

Variables	AUC (95% CI)	*p* value	Cut-off point	Sensitivity	Specificity
HOMA-IR index	0.627	0.001	1.215	0.836	0.429

Abbreviations: ROC curve, receiver operating characteristic curve; CI, confidence interval.

## Abbreviations

HOMA-IR	homeostasis model assessment of insulin resistance
IR	insulin resistance
CVD	cardiovascular disease
CKD	chronic kidney disease
WC	waist circumference
FRS	Framingham risk score
BMI	body mass index
BP	blood pressure
FPG	fasting plasma glucose
HDL-C	high-density lipoprotein cholesterol
LDL-C	low-density lipoprotein cholesterol
TG	triglyceride
ALT	alanine aminotransferase
eGFR	estimated glomerular filtration rate
ROC	receiver operating characteristic
AUC	area under the ROC curve
OR	odds ratio
aOR	adjusted odds ratio
